# Patient-Reported Experiences of Home-Based Breathing Exercises After Cardiac Surgery: A Prospective Questionnaire-Based Cohort Study

**DOI:** 10.1177/23743735251348849

**Published:** 2025-06-04

**Authors:** Elisabeth Westerdahl, Charlotte Urell, Henrik Johansson, Barbara Cristina Brocki, Marcus Jonsson

**Affiliations:** 1 University Health Care Research Center, Faculty of Medicine and Health, 6233 Örebro University, Örebro, Sweden; 2 Department of Women's and Children's Health, Physiotherapy, 8097 Uppsala University, Uppsala, Sweden; 3 Department of Physiotherapy and Occupational Therapy, 53141 Aalborg University Hospital, Aalborg, Denmark; 4 Department of Physiotherapy, Faculty of Medicine and Health, 6233 Örebro University, Örebro, Sweden

**Keywords:** breathing exercises, physical therapy, postoperative, rehabilitation, thoracic surgery

## Abstract

This study assessed patient-reported experiences and perceived respiratory outcomes of home-based deep breathing exercises 3 months after cardiac surgery. A postal questionnaire was sent to 120 patients at a Swedish university hospital, with 92 respondents (77%). The majority (89%) performed breathing exercises after discharge, with 77% reporting them as very easy to perform. Most patients continued the exercises for 2 to 4 weeks (41%) or 1 to 2 months (20%), typically practicing 3 times per day. The exercises were well tolerated, with 87% experiencing no discomfort. Motivation was generally high—43% felt very motivated, and 78% found the exercises useful. While chest pain was reported as low and 56% experienced improved breathing, nearly half of the respondents reported difficulties with coughing, and 10% sought medical care for respiratory infections. Overall, home-based deep breathing exercises were perceived as beneficial and well accepted. However, engagement and adherence varied, highlighting the need for tailored support to encourage sustained participation.

## Introduction

Cardiac surgery is performed for coronary artery bypass grafting, valve replacement, aortic reconstruction, and other cardiovascular deficits. The surgical procedure, which includes opening of the thoracic cage, may cause postoperative pain, restrictive pulmonary impairments, and decreased oxygenation in the early postoperative period. Reasons for the pulmonary impairment are multifactorial, and include the influence of anesthesia, mechanical ventilation, extracorporeal circulation, surgical incision, and medication. Postoperative pulmonary complications following cardiac surgery are a significant concern and a major contributor to morbidity and mortality.^1,2^ Pneumonia remains the most prevalent hospital-acquired infection after cardiac surgery, despite the use of preventive strategies.^3,4^ The first few days after surgery constitute the most vulnerable period, with reduced lung volumes that then gradually improve over several months.^5,6^ Pulmonary restrictive impairments have been described to remain in some patients even 1 year after surgery.^7^ Postoperative pulmonary impairments have been associated with lower physical activity level after surgery.^8^ A reduced ability to perform activities of daily living at discharge following open heart or aortic surgery has been shown to predict mortality and readmission in elderly patients.^9^ Self-management strategies that promote and sustain an active lifestyle following surgery could possibly prevent hospital readmission.

Chest physiotherapy treatments during hospital stays are commonly provided to prevent complications after cardiac surgery, but clinical practices vary globally, and the most optimal breathing exercises remain to be determined.^10–12^ The aim of the treatment is to reduce the rate of complications, shorten the length of hospital stay, and increase health-related quality of life. Patients are often recommended to perform different kinds of deep breathing exercises, with or without a positive expiratory pressure (PEP) device, during their hospital stay.^13^ However, little has been published regarding how long after surgery the patients should continue to perform the breathing exercises, and whether it is beneficial to keep performing the exercises after discharge.^14,15^ We previously demonstrated that pulmonary function remained significantly impaired 2 months after cardiac surgery compared to preoperative values, with breathing exercises during this period showing limited impact on objectively measured pulmonary function. However, the subjective effects of these exercises were not evaluated.^15^ The effectiveness of breathing exercises performed after discharge remains unproven, and evidence-based guidelines are lacking. As a result, physiotherapists must rely on clinical judgment when prescribing these exercises, while patient-reported preferences and outcomes have yet to be adequately assessed.

The aim of this study was therefore to assess patient-reported performance and experiences of home-based postoperative deep breathing exercises, as well as subjectively reported respiratory outcomes, 3 months after cardiac surgery.

## Methods

This prospective cohort study was conducted at the Department of Cardiothoracic and Vascular Surgery, XXX University Hospital, Sweden. Participants were selected from a cohort of 122 cardiac surgery patients initially recruited for an international multicenter study of pulmonary complications (the CHESTY study; ACTRN: 12616001020471). The principal investigator (EW) approached all patients in the surgical ward, typically in the afternoon on the day before surgery, after patients had met the thoracic surgeon, to invite them to participate in the study. Patients undergoing cardiac surgery (coronary artery bypass grafting, valve surgery, and aortic surgery) via sternotomy were included. One patient did not want to participate in the present study and another patient had died, leaving 120 patients eligible for this 3-month follow-up substudy. No sample size calculation was performed for the present study. Ethical approval was obtained for the cohort study including the questionnaire follow up from the Regional Ethical Review Board in XXX (ref: 2018-329, date: 2018-08-22). All patients provided written informed consent during their in-hospital stay.

While in hospital, all study patients were treated according to regular pre-, peri-, and postoperative routines. Physiotherapy treatment was delivered daily according to standard routines, including early mobilization and postoperative deep breathing exercises performed with a PEP device (RIUM breathing exerciser; Rium Medical AB, Åkersberga, Sweden). Patients were instructed to perform 30 slow, deep breaths (3 sets of 10 breaths with short pauses) using an expiratory pressure of 10 to 15 cmH_2_O during each waking hour of their in-hospital stay.^16^ At discharge from the cardiothoracic setting, patients were individually advised by a physiotherapist to continue performing 30 deep breaths with the PEP device, 4 to 5 times a day for the first few days. Patients were instructed to perform the breathing exercises until they could take fully deep breaths and had become physically active, or otherwise to continue during the 6 to 8 week healing period following the sternotomy, as previously described.^15^

A study-specific questionnaire was sent by postal mail to the patients’ homes 3 months after surgery. The study specific questionnaire consisted of 20 questions and is presented in Table 1. The ordinal scale questions asked participants to rank their answers in a specific order or degree, providing insight into the relative intensity or frequency of their experiences or opinions. The purpose of the questionnaire was to capture postoperative experiences and adherence to breathing exercises following cardiac surgery. It assessed the perceived benefits and utility of physiotherapy advice on the breathing exercises provided to patients at discharge. Additionally, patient-reported outcomes were used to assess participants’ perceptions of their ability to breathe, issues with coughing or secretion, thoracic pain, incidence of respiratory infections, and any potential adverse effects associated with the breathing exercises. Demographic and clinical characteristics were collected from medical records.

**Table 1.** Study-Specific Questionnaire.

Question	Response Options
How would you describe your current breathing ability compared to before your surgery?	• Improved • Unchanged • Impaired
Have you experienced any difficulties with coughing or secretion management after surgery?	• No, not at all • Minor difficulties • Moderate difficulties • Major difficulties
Have you had a respiratory infection or pneumonia in the past 3 months following your surgery?	• No • Yes
Have you sought medical care for a respiratory infection or pneumonia after the operation?	• No • Yes
In the past 3 months, have you used any of the following medications?—Antibiotics/penicillin for respiratory infection or pneumonia	• No • Yes
In the past 3 months, have you used any of the following medications?—Medications for obstructive lung disease/COPD/asthma	• No • Yes
Have you experienced any pain in your chest or thoracic cage during the past week?	• No, never • Yes, sometimes • Yes, constantly
Which activity is the most painful?	Open-ended response
How would you assess your chest pain at rest in a sitting position?	Numeric Rating Scale (0-10)
How would you assess your chest pain during coughing?	Numeric Rating Scale (0-10)
Did you meet with or receive treatment from a physiotherapist at the hospital?	• No • Yes
Did you practice breathing exercises with a breathing device after surgery?	• No • Yes
Was the breathing device technique easy to perform?	• Yes, very easy to perform • Yes, quite easy to perform • No, it was difficult to perform
For how long after surgery did you continue performing daily breathing exercises with the device?	• Only at the hospital • At home for 1 week • At home for 2-4 weeks • At home for 1-2 months • Still practicing at 3 months postoperatively
If you practiced breathing exercises at home, how often did you perform them each day?	Open-ended response

### Statistical Analysis

Descriptive statistics were used to summarize and describe the characteristics and postoperative experiences of the sample. Frequency and percentage were calculated for categorical variables (eg, patient-reported motivation, perceived benefits and discomfort related to breathing exercises, incidence of respiratory infections), while mean, standard deviation (SD), median (Md), interquartile range (IQR), number, percentage, and range (min-max) were calculated for continuous variables (eg, chest pain intensity, frequency of breathing exercises). Analyses were performed in version 29.0 of the SPSS software package (IBM, Armonk, NY, USA).

## Results

In total, 92 patients (response rate: 77%) returned the follow-up survey 3 months after cardiac surgery. Baseline demographics, clinical characteristics, and surgical data are presented in Table 2. Seven participants (8%) reported use of medication for chronic obstructive pulmonary disease and/or asthma during the 3-month postoperative period.

**Table 2.** Patient Characteristics and Surgical Data for Cardiac Surgery patients.

	Cardiac surgery patients (*n* = 92)
Age, years	69 ± 8
Gender, male	75 (82%)
Weight, kg	85 ± 17
Height, cm	175 ± 9
BMI, kg/m^2^	28 ± 5
Obesity (BMI >30)	25 (27%)
Diabetes mellitus	26 (28%)
Chronic renal failure	1 (1%)
Smoker/ever smoked	48 (52%)
Current smoker	7 (8%)
Smoking, packs per year	21 ± 24
Asthma	7 (8%)
COPD/ILD	12 (13%)
Isolated CABG surgery	36 (39%)
Isolated valve/valves surgery	31 (34%)
CABG and valve surgery	19 (21%)
Aortic surgery	6 (6%)
ASA class 1-2: healthy/mild systemic disease	7 (7%)
ASA class 3-4 severe systemic disease^a^	67 (73%)
Surgery, elective	73 (80%)
Surgery, emergency or expedited	19 (20%)
Operation time, h	6 ± 1

Abbreviations: ASA, American Society of Anesthesiologists physical status classification; BMI, body mass index; CABG, coronary artery bypass grafting; COPD, chronic obstructive pulmonary disease; ILD, interstitial lung disease.

^a^ Missing values: *n* = 18 (20%).

Data are presented as mean ± standard deviation or *n* (%).

Three months after discharge from the hospital, 89% (*n* = 82) of the patients reported having practiced breathing exercises following discharge. Regarding the duration of daily breathing exercises, 7% (*n* = 6) of the patients performed the exercises only while at the hospital, while 18% (*n* = 17) continued the exercises at home for 1 week, 41% (*n* = 38) for 2 to 4 weeks, and 20% (*n* = 18) for 1 to 2 months. Additionally, 10% (*n* = 9) of patients were still practicing breathing exercises at home 3 months postoperatively. The rate of missing data on this question was 4% (*n* = 4). In terms of frequency of performing the deep breathing session (3 × 10 deep breaths) setup at home, patients reported practicing an average of 3 ± 1 times per day (range: 1-5).

Of the total group, 77% (*n* = 71) found the breathing exercise technique very easy to perform, 14% (*n* = 13) found it quite easy to perform, 4% (*n* = 3) found it difficult to perform and 5% (*n* = 5) did not answer this question. The patients’ responses regarding their motivation to carry out home-based breathing exercises, the perceived benefit of the exercises, and the level of discomfort experienced are summarized in Table 3.

**Table 3.** Patient Responses on Motivation, Perceived Benefit, and Discomfort Related to Breathing Exercises Performed at Home Following Cardiac Surgery.

	Percentage of patients, % (*n*)
Motivation to carry out breathing training	
Not at all motivated	3% (*n* = 3)
Somewhat motivated	51% (*n* = 47)
Very motivated	43% (*n* = 39)
Missing values	3% (*n* = 3)
Perceived benefit from breathing exercises	
Very useful	30% (*n* = 28)
Somewhat useful	48% (*n* = 44)
No perceived benefit	14% (*n* = 13)
Missing values	8% (*n* = 7)
Discomfort associated with breathing exercises	
Significant discomfort	0% (*n* = 0)
Some discomfort	10% (*n* = 9)
No discomfort	87% (*n* = 80)
Missing values	3% (*n* = 3)

Percentages represent the proportion of patients in each category, with missing values indicated where applicable.

When asked to compare their experiences, comparing their current breathing ability to their presurgery state, 56% (*n* = 52) of the patients reported it as improved, 37% (*n* = 34) reported it as unchanged, and 7% (*n *= 6) reported it as impaired. Improvement was most often related to it being “easier to breathe during exertion,” while impairment was most often related to dyspnea and difficulty performing deep breathing.

Concerning coughing or secretion problems 3 months after surgery, 53% (*n* = 49) of patients experienced no difficulties, 28% (*n* = 26) had minor difficulties, 13% (*n* = 12) had moderate difficulties, and 6% (*n* = 5) had major difficulties.

Overall, 8% (*n* = 7) of patients reported having a respiratory infection or pneumonia in the 3-month period following surgery. Moreover, 10% (*n* = 9) of patients had sought medical care for a respiratory infection or pneumonia after the surgery, and 5% (*n* = 5) had used antibiotics/penicillin for a respiratory infection/pneumonia in the past 3 months.

When asked about pain in the chest/thoracic cage during the past week, 65% (*n* = 60) reported no pain, 29% (*n* = 26) reported occasional pain, 2% (*n* = 2) reported constant pain, and 4% (*n* = 4) did not answer. Overall, the reported chest pain intensity 3 months postoperatively was low: at rest in a sitting position, the median pain score was 0 (IQR: 0-0), and during coughing, it was 1 (IQR: 0-2). Activities mentioned as the most painful were coughing, deep breathing, getting in and out of bed, turning over in bed, lifting the arms, and leaning forward while exercising.

## Discussion

This prospective cohort study provides insights into patient-reported outcomes and experiences of postoperative breathing exercises during the first 3 months after cardiac surgery. The majority (89%) of patients engaged in breathing exercises after hospital discharge, indicating adherence to the prescribed regimen. To our knowledge, no prior research has explored this aspect of recovery.

There was a notable variation in the duration of breathing exercise practice after surgery. While 6% of patients ceased exercises upon leaving the hospital, a significant proportion continued for extended periods, with 20% practicing for 1 to 2 months and 10% still engaged in deep breathing exercises 3 months postoperatively. The average reported frequency of exercises was 3 times per day. However, as personalized recommendations regarding the duration and frequency of breathing exercises were provided at discharge based on standard routines at the cardiothoracic department, and we did not have data on these individual recommendations, it was not possible to assess adherence in terms of duration and frequency.

Most patients (77%) found the exercises very easy to perform, with only a small fraction (4%) reporting difficulty. This suggests that the exercises are generally manageable for patients to perform at home. Motivation to perform breathing exercises was generally high, with 43% of patients feeling very motivated. Patients’ appreciation of the breathing exercises is an important issue for maintenance of exercise performance.

The perceived benefits were also significant, with 78% of patients finding the exercises either somewhat useful or very useful. A small percentage (14%) reported no perceived benefit. No previous studies have assessed how patients perceive the value of postoperative advice on breathing exercises after discharge, despite the common recommendation to continue these exercises at home.

Breathing exercises are commonly recommended after cardiac surgery, and are often combined with mobilization during the in-hospital phase.^17–19^ Altered respiratory movements and restrictive pulmonary function loss for several months after surgery have been demonstrated,^5,20^ but a previous study by our group was not able to verify the effectiveness of specific breathing exercises following discharge.^15^ There is considerable global variability in clinical practice regarding prescription of physiotherapy and advice on different breathing techniques after surgery. In this study, we chose to evaluate deep breathing exercises that are commonly used in clinical practice in Sweden.^13^ It is important to emphasize the value of providing tailored education on breathing exercises, particularly given the heterogeneity of surgical procedures in our patient cohort. Customized instruction may enhance postoperative outcomes by addressing the specific needs and limitations associated with particular patient subgroups. The role of self-management and patient preferences warrants further exploration, particularly from a qualitative perspective. While qualitative data specific to cardiac surgery is limited, research from other patient groups highlights the importance of family and friends in the recovery phase.^21^ Integrating such perspectives into future research could help tailor postoperative support more effectively.

More than half of the patients in the present study (56%) reported improved breathing ability 3 months after surgery compared to their preoperative condition. The improvement was primarily associated with easier breathing during exertion, suggesting that the patients experienced enhanced functional capacity. However, 7% of patients reported impaired breathing, mainly due to dyspnea and difficulties in taking deep breaths; this highlights a subset of patients who may require continuing support or treatment after hospital discharge.

Following surgery, pain from the sternotomy incision may affect postoperative recovery.^22^ Restrictive precautions, such as limited use of the arms, are often prescribed to prevent sternal complications.^23^ In the present study, chest pain 3 months after surgery was predominantly reported as minimal, with 65% of patients experiencing no pain and only a small fraction (2%) suffering constant pain. However, specific activities such as coughing, deep breathing, and physical movements like getting in and out of bed were identified as painful, making the maintenance of breathing exercises after cardiac surgery challenging.^22^

The incidence of self-reported respiratory infections or pneumonia within 3 months of surgery was 8%, and 5% of the patients reported that they had required antibiotics. The finding that some patients continue to experience breathing difficulties and complications up to 3 months following surgery indicates the need for extended support and follow-up to address persistent issues such as coughing, secretion problems, and postoperative pulmonary infections.

Postoperative pulmonary complications are relatively common after cardiac surgery, with prevalence estimates ranging widely (from about 10% to 70%) depending on the criteria used and patient demographics. During hospital stay a prevalence of postoperative pulmonary complications of 55% to 69% has been reported.^24,25^ Characteristics that have been associated with mortality and complications following cardiac surgery are advanced age, severity of heart failure, emergent surgery, higher BMI, preoperative renal failure, chronic obstructive pulmonary disease, and impaired oxygenation at discharge.^26^ Postoperative pulmonary impairment after discharge following cardiac surgery may reduce the patient's ability to be physically active several months after surgery.^7,9^ A low activity of daily life status at hospital discharge after cardiac and aortic surgery has been shown to predict mortality and readmission in elderly patients.^9^ The potential benefits of breathing exercises in selected patients may warrant further exploration and longer follow-up is needed to assess the sustained benefits and potential late-onset concerns.

A total of 92 patients participated in the follow-up survey, providing a comprehensive overview of their recovery and the impact of breathing exercises on their postoperative outcomes. The response rate was good (77%), considering that the questionnaire was only sent once. A follow-up survey, designed to assess patient recovery and the impact of breathing exercises after surgery, was completed by 92 participants, representing a 77% response rate. This response rate is considered acceptable, especially given that the survey was distributed only once. However, several limitations must be acknowledged.

First, the data collected relied on patient self-reporting, which can introduce recall bias. Patients may not accurately remember their experiences or may overestimate their adherence to prescribed exercises. Second, the study was conducted at a single hospital specializing in cardiothoracic surgery. This single-center design limits the ability to generalize the findings to other patient populations or healthcare settings. Third, the analysis did not control for potentially influential factors such as the specific type and complexity of surgery, preexisting health conditions, or other postoperative treatments. This lack of control makes it difficult to isolate the effects of breathing exercises alone. Finally, the study did not include enough participants to allow for the analysis of how breathing exercises may have affected patients differently based on the type of surgery they underwent.

To enhance the effectiveness of postoperative breathing exercises, future research should prioritize several key areas. Understanding and addressing individual patient needs, preferences, and barriers to adherence is crucial for improving at-home exercise compliance. This includes tailoring education strategies to meet diverse learning styles and addressing potential obstacles to participation. Research by Akyüz et al^27^ indicates that a significant portion of patients report inadequate information regarding preoperative breathing exercises, favoring simpler, more direct instructions. This underscores the need for improved educational strategies, specifically focusing on providing clear and concise guidance on postsurgical expectations and discharge care to enhance patient understanding and compliance. Future research should focus on determining the optimal parameters for breathing exercises, including duration, frequency, and performance techniques. Identifying the most effective educational methods for conveying these parameters is also vital. Finally, strategies to augment patient motivation and adherence to breathing exercises should be investigated. This includes exploring interventions that foster sustained engagement and promote long-term compliance. By addressing these areas, future studies can contribute to the development of more effective and patient-centered postoperative care.

## Conclusion

Overall, the findings from this study highlight the positive subjective impact of deep breathing exercises on postoperative recovery in cardiac surgery patients. The high adherence rates, perceived benefits, and low discomfort levels suggest that these exercises should continue to be a recommended component of postoperative care. However, the persistent issues concerning coughing and secretion problems and reporting of postoperative pulmonary infections indicate a need for further approaches and potentially extended support for optimal recovery.
